# Perceived Stress and Smartphone Addiction in Medical College Students: The Mediating Role of Negative Emotions and the Moderating Role of Psychological Capital

**DOI:** 10.3389/fpsyg.2021.660234

**Published:** 2021-07-21

**Authors:** Wenbo Wang, Anam Mehmood, Ping Li, Zhaonan Yang, Jinbao Niu, Haiyun Chu, Zhengxue Qiao, Xiaohui Qiu, Jiawei Zhou, Yanjie Yang, Xiuxian Yang

**Affiliations:** Psychology Department of the Public Health Institute of Harbin Medical University, Harbin, China

**Keywords:** perceived stress, smartphone addiction, medical college students, psychological capital, negative emotion

## Abstract

**Background**: Many studies have confirmed the existence of an extremely close relationship between smartphone addiction and perceived stress. However, the mediating and moderating mechanisms underlying the association between perceived stress and smartphone addiction in medical college students remain largely unexplored.

**Methods**: A questionnaire was distributed among a total of 769 medical college students in Heilongjiang Province, China. Participants completed measures of perceived stress, smartphone addiction, negative emotions, and psychological capital. Pearson’s correlation analysis was used to test the correlations between variables. The analysis of a moderated mediation model was performed using Hayes’s PROCESS macro.

**Results**: Pearson’s correlation analysis indicated that perceived stress (*r* = 0.18, *p* < 0.01) and negative emotions (*r* = 0.31, *p* < 0.01) were positively correlated with smartphone addiction, and psychological capital was negatively correlated with smartphone addiction (*r* = −0.29, *p* < 0.01). The moderated mediation analysis indicated that negative emotions partially mediated the association between perceived stress and smartphone addiction [mediation effect accounted for 33.3%, *SE* = 0.10, 95% *CI* = (0.10, 0.24)], and the first stage of the mediation process was significantly moderated by psychological capital [moderated mediation = −0.01, *SE* = 0.01, 95% *CI* = (−0.01, −0.00)].

**Conclusion**: Negative emotions play a mediating role between perceived stress and smartphone addiction, and psychological capital plays an important moderating role in the first stage of the mediation process.

## Introduction

The smartphone has become an important tool for accessing information, interaction, and entertainment in modern society. However, excessive use of smartphones has accumulating adverse influences on individuals, which has aroused great concern within society. Smartphone addiction, also known as problematic smartphone use or mobile phone dependence (Gao et al., 2017), is essentially behavioral addiction. It refers to the individual behavior being out of control because of the use of mobile phones, resulting in a state of obsession (Gao et al., 2017). The studies reported that smartphone addiction in adolescence ranges from 20 to 40%. Especially, a recent Filipino study found that the incidence of smartphone addiction is as high as 60% (Buctot et al., 2020). The individual’s physiological, psychological, and social functions are significantly impaired (Yen et al., 2009). Specifically, a large number of studies have shown that smartphone addiction can cause a variety of maladjustment, including physical health difficulties (Kim et al., 2015), sleep disturbances (Liu et al., 2017), academic failures (Samaha and Hawi, 2016), and emotional and behavioral problems (Chen et al., 2016, 2020).

The general strain theory is originally used for explaining criminal behaviors (Agnew, 1992), and now it has been well used for analyzing addictive behaviors (Eitle et al., 2013; Jun and Choi, 2015). According to the general strain theory, strains or stressors increase the likelihood of negative emotions like anger and frustration (Agnew, 2001). These emotions cause corrective behavior (Agnew, 2001), and addictive behavior may be a method for reducing strain or alleviating negative emotions. Medical college students, as a special group of college students, have high levels of stress that could be due to academic burden, frequency of examinations, lengthy academic curriculum, and worrying about the future (Gazzaz et al., 2018). When faced with this stress, they are more likely to use smartphones as a way to relieve stress (Yang et al., 2020). Perceived stress refers to the degree to which an individual perceives an external event as stress (Cohen et al., 1984). Whether the objective stress affects the individual depends on the individual interpretation and perception of the stress event (Cohen et al., 1984). Perceived stress could make an individual believe they are in a stress situation, which is considered to be a risk factor in the occurrence and recurrence of many addictions, such as problematic online gaming (Snodgrass et al., 2014), substance abuse (Sinha, 2008; Tavolacci et al., 2013), internet addiction (Jun and Choi, 2015), and so on. Particularly, some studies have confirmed that stress could effectively predict smartphone addiction (Chiu, 2014; Kuang-Tsan and Fu-Yuan, 2017). Individuals who perceive more stress are more inclined to engage in smartphone addiction (Liu et al., 2018).

Unfortunately, previous studies have made valuable contributions to the relationship between stress and smartphone addiction; however, the mediating (i.e., how perceived stress relates to medical college students’ smartphone addiction) and moderating mechanisms (i.e., when the relation is most potent) underlying the association between perceived stress and smartphone addiction in medical college students remain largely unexplored. Confirming its mediating and moderating mechanisms could be critical to advance our understanding of smartphone addiction in medical college students and to develop an effective intervention as well.

### The Mediating Role of Negative Emotions

Abundant studies have shown that perceived stress is positively correlated with negative emotions (Spada et al., 2008; Anitei and Chraif, 2013). Stress is one of the most important risks leading to mental health problems. To some extent, the existence of stress can break the balance between the individual and the environment. The imbalance makes it difficult for the individual to adapt to the impact of objective events on themselves, which leads to some negative emotions such as anxiety and depression. Negative emotions have an important impact on individual cognition and behavior. Previous studies have found a positive correlation between negative emotions and problematic behavior. On the one hand, negative emotions can cause substance problematic use, such as drug abuse (Ketcherside and Filbey, 2015). Apart from substance problematic use, a large number of studies have shown that negative emotions are related to nonmaterial problematic use, such as internet addiction (Young and Rogers, 1998; Scimeca et al., 2014) and smartphone addiction (Ghasempour and Mahmoodi-Aghdam, 2015; Matar Boumosleh and Jaalouk, 2017). For example, one study pointed out that anxiety and depression scores emerged as the independent positive predictors of smartphone addiction (Matar Boumosleh and Jaalouk, 2017). Individuals with high depression scores are more likely to become addicted to their smartphone. Relevant studies have shown that mood regulation (defined as reducing negative feelings such as loneliness, anxiety, depression, stress) could reduce the occurrence of smartphone addiction among a convenient sample of 394 Chinese university students (Zhang et al., 2014; Stanković et al., 2021). Furthermore, some studies have indicated that individuals are prone to eliminate negative emotions accumulated in daily life through negative means including substance abuse, dependence, and addiction (Ketcherside and Filbey, 2015). Considering that smartphone addiction behavior is problematic behavior, we could speculate that negative emotions are significantly positively correlated with smartphone addiction based on the mentioned studies.

Therefore, we assume that negative emotions will play a mediating role in the relationship between perceived stress and smartphone addiction. This hypothesis could be corroborated by similar studies. For example, many researchers have found that negative emotions mediated the relationship between stress and problematic behaviors, including eating disorders (Goldschmidt et al., 2013) and problematic use of marijuana (Ketcherside and Filbey, 2015). To our knowledge, the mediated effect in the relation of perceived stress and smartphone addiction in medical college students remains largely unexplored.

### The Moderating Role of Psychological Capital

Psychological capital is an important personal resource, defined by Luthans et al. (2004) as “a positive psychological state that an individual performs in the process of growth and development.” It is composed of four psychological resource capacities, namely, self-efficacy, hope, optimism, and resilience (Luthans et al., 2004). According to Bandura’s Social Cognitive Theory (Bandura, 1986; Bandura, 2002), efficacy is defined as “having the confidence to undertake and make the necessary effort to succeed at challenging tasks”; hope means persevering toward goals and when necessary, redirecting paths to goals in order to succeed; optimism refers to “a mood or attitude”; resilience is defined as “sustaining and bouncing back and even beyond to attain success when beset by problems and adversity” (Luthans et al., 2008). Resilience could help an individual cope with stress effectively and achieve good adaptation and development (Dyrbye et al., 2010; Hu et al., 2015). The general strain theory incorporates conditioning factors into the theory to explain individual differences in adaptations to strain (Jang and Johnson, 2003). Agnew proposed that an individual’s internal and external factors condition the effects of strain on negative emotions, which in turn affects deviant coping (Agnew, 1992). That is, the conditioning factors influence an individual’s selection of deviant or non-deviant coping by decreasing or increasing the likelihood that the individual will experience negative emotions in response to strain. For example, an angry adolescent high in self-efficacy is less likely to turn to delinquency than an equally angry adolescent low in self-efficacy (Jang and Johnson, 2003). Particularly, some studies have revealed the moderating effect of resilience (Wingo et al., 2010). Wingo et al. (2010) found that resilience moderated depressive symptom severity in a cross-sectional study of 792 adults; that is, individuals high in resilience had lower levels of depression. In a survey of Chinese physicians, psychological capital moderates the association between occupational stress and depressive symptoms in female physicians. Psychological capital could be a positive resource for combating depressive symptoms (Shen et al., 2014). To our knowledge, no studies have examined psychological capital as a moderator of the direct and/or indirect associations between perceived stress and smartphone addiction.

**Figure 1:**
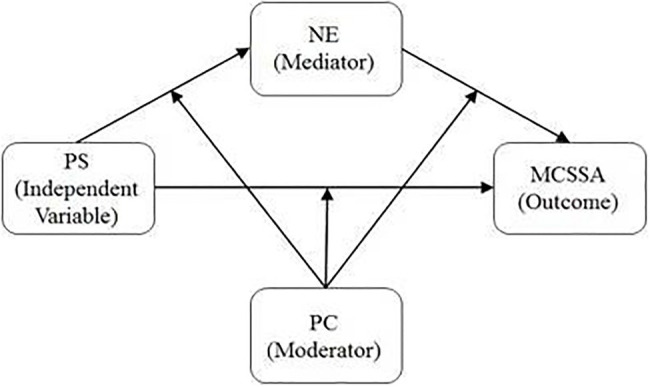
The proposed moderated mediation model. PS, perceived stress; NE, negative emotion; PC, psychological capital; MCSSA, medical college student smartphone addiction.

Based on the literature reviewed above, we put forward the following hypotheses:

*Hypothesis* 1: Negative emotions will mediate the link between perceived stress and smartphone addiction in medical college students.
*Hypothesis* 2: The direct and/or indirect associations between perceived stress and smartphone addiction *via* negative emotions will vary as a function of psychological capital. Figure 1 illustrates the proposed model.

## Materials and Methods

### Participants

An online survey including the purpose of the study and an informed consent form was used to collect data in Harbin Medical University, China. The online survey was disseminated using a hyperlink and a QR code. Only after providing informed consent could participants continue with the online survey. A total of 769 students were recruited using a convenience sampling method, with an effective rate of 81%.

The mean age of the participants was 20.46 years (*SD* = 1.40, range = 17–29 years). Of the participants, 19% were men, and 81% of the participants were women. Of the participants, 467 (61%) were from cities and 302 (39%) were from rural areas. Finally, 58% of medical college students came from one-child families, and 42% of them came from non-only child families.

### Measures

#### Mobile Phone Addiction Index Scale

Smartphone addiction was measured by the Mobile Phone Addiction Index Scale (Leung, 2008). The scale consists of 17 items that measure four dimensions of smartphone addiction: inability to control cravings, anxiety and feeling lost, withdrawal and escape, and productivity loss. Participants answered these items on a 5-point scale (ranging from 1 = never to 5 = always). Previous studies have shown that the MPAI had good reliability and validity in Chinese adolescents and young adults (Lian et al., 2016). For the current study, the measure demonstrated a good reliability (*ɑ* = 0.88).

#### The Perceived Stress Scale

Perceived stress was assessed using the Perceived Stress Scale (González-Ramírez and Hernández, 2007). This scale consists of 14 items (e.g., “Feeling nervous and stress?”). The participants rated each item on a 5-point scale ranging from 1 = never to 5 = very much, with higher scores indicating a higher level of perceived stress. For the current study, the measure demonstrated a good reliability (*ɑ* = 0.82).

#### The Positive and Negative Affect Scale

Negative emotions were measured by the Positive and Negative Affect Scale (Watson et al., 1988). The scale consists of 20 items that measure two dimensions of emotions: positive emotions and negative emotions. This scale was measured in a 5-point scale (ranging from 1 = almost none to 5 = very much), with higher negative emotional scores indicating individuals were puzzled and in pain. The survey asked medical students to tick behind each adjective according to the actual situation in the last week. There were five options after each adjective: almost none, less, medium, more, and very much. The Cronbach’s ɑ in this study was 0.88.

#### Positive Psychological Capital Questionnaire

The Psychological Capital Questionnaire consists of 26 items, and there are four dimensions: self-efficacy, resilience, hope, and optimism (Luthans et al., 2007). There are seven items for the self-efficacy dimension, seven items for the resilience dimension, six items for the hope dimension, and six items for the optimistic dimension. This questionnaire was measured on a 7-point scale (ranging from 1 = not at all to 7 = completely suitable), with higher scores indicating that medical college students had a higher psychological capital level. The Cronbach’s ɑ in this study was 0.84.

### Procedure

The data were collected in university classrooms between April and May 2017. Trained postgraduate students administered the measures using scripts and a manual of procedures to ensure the standardization of the data collection process. Informed consent was obtained from college students before the data collection. Students were informed that their participation was completely voluntary, and they could decline participation at any time. Participants received a gift as an incentive after they completed all questionnaires.

### Statistical Analysis

All statistical analyses were conducted using SPSS 23.0. First, descriptive statistics (i.e., M, SD) were calculated for all variables, followed by bivariate associations among these variables. Second, we followed MacKinnon’s four-step procedure to establish a mediation effect (MacKinnon and Tofighi, 2012). Third, we further examined whether the mediation process was moderated by psychological capital. Moderated mediation is often used to examine whether the magnitude of a mediation effect is conditional on the value of a moderator (Muller et al., 2006). The analysis of the moderated mediation model was performed using Hayes’s PROCESS macro (Model 59; Hayes, 2013). All continuous variables were standardized, and the interaction terms were computed from these standardized scores. In addition, the bootstrapping method was applied to examine the significance of all the effects to obtain robust standard errors for parameter estimation (Hayes, 2013). The bootstrapping method produces 95% bias-corrected confidence intervals of these effects from 1,000 resamples of the data. Confidence intervals that do not include zero indicate the effects that are significant.

## Results

### Preliminary Analyses

The incidence of mobile phone addiction was 17.8 and 19.1% in male and female students, respectively. The gender differences in the incidence were not significant in our study. Means, SD, and correlations for all variables are presented in Table 1. Correlation analyses showed that perceived stress was positively associated with smartphone addiction, *r* = 0.18, *p* < 0.01, indicating that perceived stress was a risk factor for smartphone addiction in medical college students. Psychological capital was negatively associated with smartphone addiction, *r* = −0.29, *p* < 0.01. In addition, negative emotions were positively related to smartphone addiction, *r* = 0.31, *p* < 0.01, indicating that medical college students with high negative emotions were more likely to become addicted to their smartphone. Finally, perceived stress was positively related to negative emotions, *r* = 0.20, *p* < 0.01.

**Table 1:** Means, standard deviations, and correlations of the main study variables.

Variable	1	2	3	4	5	6	7	8
1. Gender	—							
2. Age	−0.13^*^	—						
3. Only child	0.11^*^	0.01		—				
4. Home location	0.04	0.13^*^	0.46^*^		—			
5. Perceived stress	−0.01	−0.07	0.01	0.01	—			
6. Negative emotions	−0.03	0.06	−0.02	0.00	0.20^*^	—		
7. Psychological capital	0.15^*^	−0.05	−0.01	−0.04	0.03	−0.42^*^	—	
8. Smartphone addiction	−0.04	0.05	0.04	0.05	0.18^*^	0.31^*^	−0.29^*^	—
M	1.81	20.5	1.42	1.39	31.96	22.14	123.55	39.08
SD	0.40	1.40	0.50	0.49	3.45	6.31	16.73	9.89

*N* = 769. SD, standard deviation.

* *p* < 0.01.

### Testing for Mediation Effect

In Hypothesis 1, we anticipated that negative emotions would mediate the relationship between perceived stress and smartphone addiction in medical college students. To test this hypothesis, we followed MacKinnon’s four-step procedure to establish the mediation effect (MacKinnon and Tofighi, 2012), which requires (a) a significant relation between perceived stress and smartphone addiction in medical college students; (b) a significant association between perceived stress and negative emotions; (c) a significant relation between negative emotions and smartphone addiction after controlling for perceived stress; and (d) a significant coefficient for the indirect path between perceived stress and smartphone addiction through negative emotions. The bias-corrected percentile bootstrap approach determines whether the last condition is satisfied.

Regression analyses indicated that in the first step, perceived stress positively predicted smartphone addiction in medical college students, *b* = 0.18, *p* < 0.01 (see Model 1 of Table 2). In the second step, perceived stress positively predicted negative emotions, *b* = 0.20, *p* < 0.01 (see Model 2 of Table 2). In the third step, when we controlled for perceived stress, negative emotions significantly and positively predicted smartphone addiction, *b* = 0.31, *p* < 0.01 (see Model 3 of Table 2). Finally, the bias-corrected percentile bootstrap method indicated that the indirect effect of perceived stress on smartphone addiction through negative emotions was significant, *ab* = 0.06, *SE* = 0.10, 95% *CI* = [0.10, 0.24]. The mediation effect accounted for 33.3% of the total effect. Overall, the above four criteria for establishing the mediation effect were fully satisfied. Therefore, Hypothesis 1 was supported.

**Table 2:** Testing the mediation effect of perceived stress on smartphone addiction.

Predictors	Model 1 (MCSSA)		Model 2 (NE)		Model 3 (MCSSA)	
	b	t	b	t	b	t
S	0.18	5.09^**^	0.20	5.63^**^	0.12	3.55^**^
NE					0.31	9.08^**^
R2	0.03		0.04		0.10	
F	25.88^**^		31.73^**^		82.42^**^	

*N* = 769. Each column is a regression model that predicts the criterion at the top of the column. PS, perceived stress; NE, negative emotions; MCSSA, medical college student smartphone addiction.

** *p* < 0.01.

### Testing for Moderated Mediation

As noted, Hypothesis 2 predicted that psychological capital would moderate the direct and/or indirect associations between perceived stress and smartphone addiction *via* negative emotions. To examine this hypothesis, we used the PROCESS macro (Model 59) developed by Hayes to test for moderated mediation (Hayes, 2013). Specially, we estimated the parameters for three regression models. In Model 1, we estimated the moderating effect of psychological capital on the relation between perceived stress and smartphone addiction in medical college students. In Model 2, we estimated the moderating effect of psychological capital on the relation between perceived stress and negative emotions. In Model 3, we estimated the moderating effect of psychological capital on the relation between negative emotions and smartphone addiction. The specifications of the three models are shown in Table 3.

**Table 3:** Testing the moderated mediation effect of perceived stress on medical college student smartphone addiction.

Predictors	Model 1 (MCSSA)		Model 2 (NE)		Model 3 (MCSSA)	
	b	t	b	t	b	t
PS	0.18	5.43^**^	0.20	6.39^**^	0.14	4.18^**^
PC	−0.29	−8.65^**^	−0.42	−13.21^**^	−0.21	−5.77^**^
PS × PC	−0.04	−1.21	−0.07	−2.04^*^	−0.03	−0.75
NE	—	—	—	—	0.19	4.88^**^
NE × PC	—	—	—	—	−0.03	−0.72
R2	0.12	—	0.22	—	0.15	—
F	35.40^**^	—	73.80^**^	—	27.01^**^	—

*N* = 769. Each column is a regression model that predicts the criterion at the top of the column. PS, perceived stress; NE, negative emotion; PC, psychological capital; MCSSA, medical college student smartphone addiction.

* *p* < 0.05;

** *p* < 0.01.

Moderated mediation was established if either or both of the two patterns existed (Muller et al., 2006; Hayes, 2013): (a) the path between perceived stress and negative emotions was moderated by psychological capital (first-stage moderation), and/or (b) the path between negative emotions and smartphone addiction was moderated by psychological capital (second-stage moderation).

**Figure 2:**
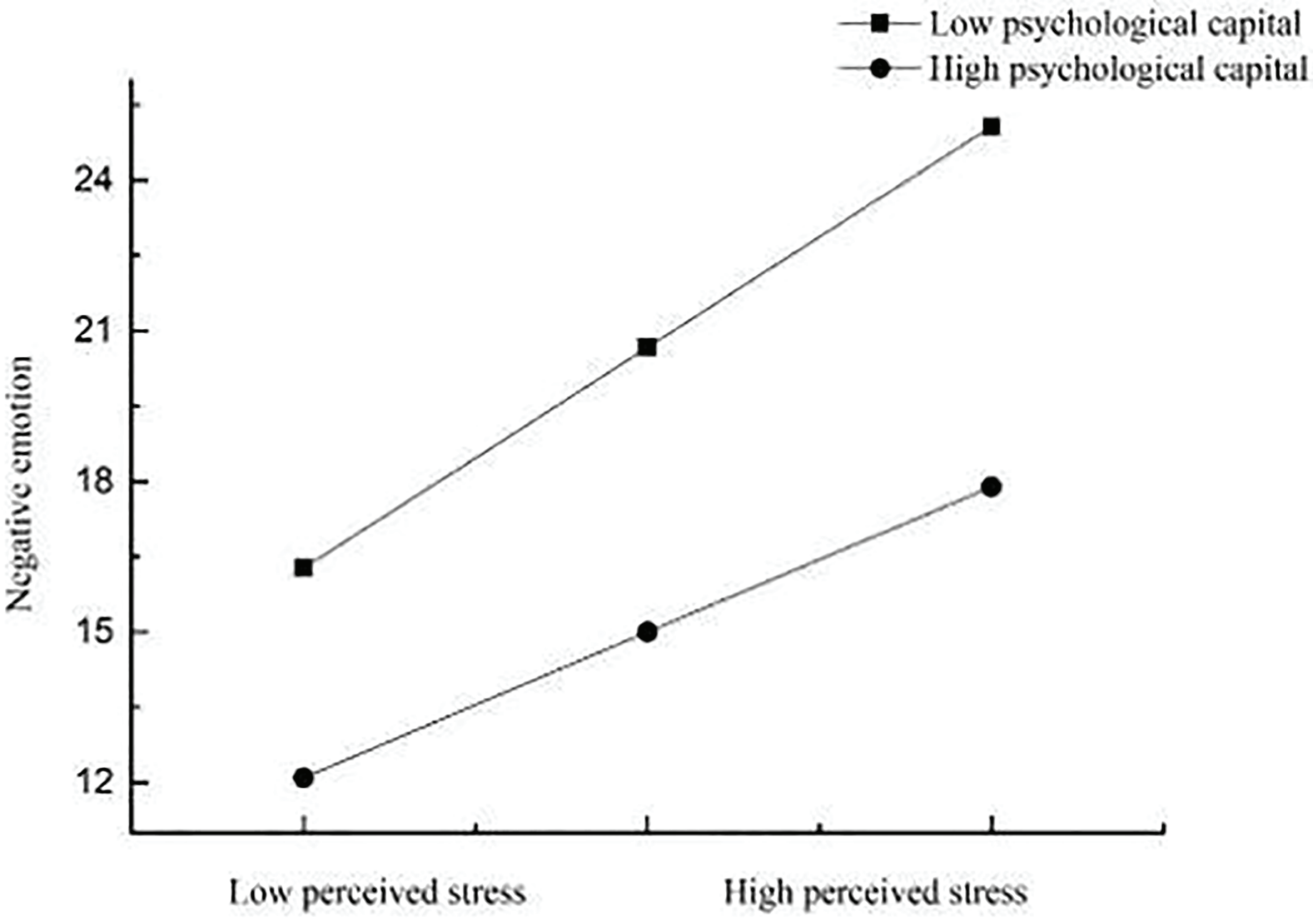
Plot of the relationship between perceived stress and negative emotions at two levels of psychological capital.

As Table 3 illustrates, in Model 1, there was a significant effect of perceived stress on smartphone addiction, *b* = 0.18, *p* < 0.01, but this effect was not moderated by psychological capital, *b* = −0.04, *p* > 0.05. Model 2 showed that the effect of perceived stress on negative emotions was significant, *b* = 0.20, *p* < 0.01, and more importantly, this effect was moderated by psychological capital, *b* = −0.07, *p* < 0.05. For descriptive purposes, we plotted the predicted negative emotions against perceived stress, separately for low and high levels of psychological capital (1 SD below the mean and 1 SD above the mean, respectively; Figure 2). Simple slope tests indicated that for medical college students with high levels of psychological capital, perceived stress was not significantly associated with negative emotions, *b_simple_* = 0.145, *p* = 0.15. However, for medical college students with low levels of psychological capital, perceived stress was significantly associated with negative emotions, *b_simple_* = 0.22, *p* < 0.05. That is, in the low psychological capital group, perceived stress has a significant positive predictive effect on negative emotions. This shows that the influence of perceived stress on negative emotions decreases with the increase in psychological capital. In other words, the indirect influence of perceived stress on smartphone addiction through negative emotions decreases with the increase in psychological capital. Model 3 indicated that there was a significant effect of negative emotions on smartphone addiction, *b* = 0.19, *p* < 0.01, but this effect was not moderated by psychological capital, *b* = −0.03, *p* > 0.05.

The bias-corrected percentile bootstrap method further indicated that the indirect effect of perceived stress on smartphone addiction through negative emotions was moderated by psychological capital, with the index of moderated mediation *b* = −0.01, *SE* = 0.01, 95% *C*I = [−0.01, −0.00]. For medical college students low in psychological capital, perceived stress had an adverse impact on smartphone addiction through increased negative emotions, *b* = 0.16, *SE* = 0.05, 95% *CI* = [0.08, 0.27]. In contrast, the indirect effect was much weaker for medical college students high in psychological capital, *b* = 0.06, *SE* = 0.03, 95% *CI* = [0.02, 0.13]. Thus, Hypothesis 2 was partially supported.

## Discussion

The incidence of smartphone addiction in our study is lower compared to other studies. The reason for this result is that there are more female subjects in this study. Precious studies reported that men are more likely to be addicted to smartphones than women (Chiang et al., 2019; Su et al., 2019; Buctot et al., 2020).

The influence of perceived stress on smartphone addiction has begun to gain empirical support (Chiu, 2014; Yang et al., 2020). However, questions concerning the underlying mediating and moderating mechanisms remain largely unknown. Our study adds to the literature by examining the mediating role of negative emotions and the moderating role of psychological capital in the relationship between perceived stress and smartphone addiction. The results indicated that the impact of perceived stress on smartphone addiction can be partially explained by negative emotions. That is, perceived stress could positively predict negative emotions, and negative emotions could positively predict smartphone addiction. Furthermore, this indirect relation was moderated by psychological capital in the first stage of the mediation process. The following sections will discuss each of the hypotheses according to the research results.

### The Mediating Role of Negative Emotions

In line with previous studies (Chiu, 2014; Kuang-Tsan and Fu-Yuan, 2017; Liu et al., 2018), we found that perceived stress was positively correlated with smartphone addiction in medical college students. The more stress medical college students perceived, the more likely they were to become addicted to their smartphone. This is consistent with the general strain theory (Jun and Choi, 2015) proposing that all kinds of strain or stress experienced by an individual would cause negative emotions, which subsequently causes problem behaviors. Many studies have shown that stress is an important risk factor for individual addictive behaviors (Lam et al., 2009; Li et al., 2010; Mai et al., 2012). For example, Mai et al. (2012) found that stress is positively associated with adolescent internet addiction. These findings suggest that stress plays an important role in addictive behavior.

In addition, our study showed that negative emotions partially mediated the link between perceived stress and smartphone addiction. For the first stage of the mediation process (i.e., the links between perceived stress and negative emotions), we revealed that negative emotions increased with perceived stress. Our result supports the views of Coyne and Downey (1991). They believed that stressors could cause emotional changes, and these stressors mainly come from the various life events experienced in daily life (i.e., study, peers, and family for university students). These multiple stressors could lead to negative emotions in medical college students. Moreover, some studies have shown that perceived stress is a strong predictor of negative emotions (Bergdahl and Bergdahl, 2002; Spada et al., 2008; Jun and Choi, 2015). For example, Spada et al. (2008) found a positive and significant correlation between perceived stress, anxiety, and depression. For the second stage of the mediation model (i.e., the links between negative emotions and smartphone addiction), this study revealed that negative emotions were positively associated with smartphone addiction in medical college students. This finding is in line with the cognitive-behavioral model of pathological internet use proposed by Davis (2001), which postulates that addiction behavior is the result of a predisposed vulnerability including negative emotions. In addition, many studies have confirmed the relationship between negative emotions and addictive behaviors (Caplan, 2007; Gámez-Guadix et al., 2012; Özdemir et al., 2014). For example, one study showed that there is a significant positive relationship between depression and smartphone addiction and also indicated that depression is able to predict and account for smartphone addiction among students (Ghasempour and Mahmoodi-Aghdam, 2015). Furthermore, Jun and Choi (2015) found that negative emotions play a mediating role in the link between academic stress and internet addiction. Based on the above theories, it clarifies the link between perceived stress and negative emotions (Bergdahl and Bergdahl, 2002; Spada et al., 2008) as well as the link between negative emotions and smartphone addiction (Matar Boumosleh and Jaalouk, 2017; Stanković et al., 2021). In this study, we integrated these two relationships with a mediation model approach. Our study goes one step further by uncovering that individuals who perceive more stress have more negative emotions, and they are more likely to engage in smartphone addiction.

This finding has important implications for policymakers who develop means to prevent and intervene in smartphone addiction among medical college students. The results suggest that it is particularly important for counselors to deal with medical college students’ negative emotions in the context of smartphone addiction. The findings also suggest that the early prevention of stress is important in halting the development of negative emotions that subsequently influence smartphone addiction. In addition, for clinical practitioners, we not only pay attention to the symptoms of internet addicts themselves, but also stress and negative emotions.

### The Moderating Role of Psychological Capital

Our findings confirmed the moderating role of psychological capital in the indirect association between perceived stress and smartphone addiction. Specifically, we found that psychological capital attenuated the link between perceived stress and negative emotions. No significant correlation between perceived stress and negative emotions was found in medical college students with higher levels of psychological capital. However, perceived stress is more likely to lead to negative emotions in medical college students with lower levels of psychological capital. Our findings are in line with the research of Li-Feng and Hua-Li (2009), which suggests that not all people who are exposed to stressful conditions feel negative emotions to the same degree. perceived stress depends not only on the effects of stressors but also on how the individual appraises the situation – one of the students’ positive psychological states such as psychological capital may attenuate the negative effects of stress on negative emotions. Moreover, Hobfoll (1989) proposed a conservation of resources theory, and Hobfoll (2012) considered that valuable resources played a positive role in the process of the individual stress response. These resources included material, power, interpersonal relationships, and positive psychological factors (Hobfoll, 1989). This theory provides a reliable perspective to explain that psychological capital could protect individuals from the adverse effects of stress, and it is a protective factor for mental health (Collishaw et al., 2007; Wingo et al., 2010; Goldstein et al., 2013). Our results also support the opinion that individuals with higher levels of psychological capital may view stress as a controllable factor and are able to recover from stressful experiences quickly and efficiently (Fredrickson et al., 2003; Tugade and Fredrickson, 2004; Tugade et al., 2004). When faced with stress, medical college students with higher levels of psychological capital might bounce back from adversity or failure with positive psychological capacity (resilience), preserve the will to accomplish a learning task or goal (hope), have more confidence and exert greater effort in the pursuit of success (self-efficacy), and have positive expectations and attributes regarding outcomes (optimism).

This finding indicates that psychological capital is a positive personal trait and an important protective factor. It prevents individuals from being affected by perceived stress, reducing their negative emotions and the likelihood of developing smartphone addiction. Our findings suggest that we should pay attention to psychological capital when helping medical college students to deal with the negative effects of perceived stress in the future. And our findings also suggest that we should enhance students’ psychological capital in their daily life to keep them from developing addiction behavior when faced with stress.

### Limitations

Several limitations must be considered when interpreting the findings of this study. First, this study was cross-sectional and cannot infer causality. For example, smartphone addiction is used as a result variable in this study, but smartphone addiction may also have a reverse effect on risk factors (such as perceived stress, negative emotions) and protective factors (such as psychological capital). Therefore, further studies should apply longitudinal or experimental designs to confirm the causal assumptions in this study. Second, the representative of the sample may restrict the general validity of our results, because our participants were from the same university. Future research may explore the proposed model among diverse populations. Third, most of the participants were female students in our study. Therefore, the results of the present study may not generalize to other university students with more male students, other age populations such as middle school students, and people of older age. Fourth, medical college students’ self-reported data on perceived stress and smartphone addiction could not be independently verified. The understanding of each questionnaire item measuring an abstract concept varies from one participant to another. Future research should use multiple informants (e.g., parents, teachers, and peers) to collect data to ensure its accuracy.

## Conclusion

In summary, this study is among the first to uncover the mediation role of negative emotions and the moderation role of psychological capital in the relation between perceived stress and smartphone addiction. It explains how, when, and how perceived stress is related to smartphone addiction. These results deepen the work of previous studies by clarifying the mediation and moderation factors in the link between perceived stress and smartphone addiction. In this study, negative emotions serve as one potential mediation mechanism between perceived stress and smartphone addiction in medical college students. Moreover, the mediation mechanism was moderated by psychological capital, and the adverse impact of perceived stress on smartphone addiction through decreased negative emotions appears to be weaker for medical college students with higher levels of psychological capital. Our findings demonstrate the importance of the moderated mediation model in understanding the mechanism linking perceived stress and smartphone addiction in medical college students.

## Data Availability Statement

The raw data supporting the conclusions of this article will be made available by the authors, without undue reservation.

## Ethics Statement

The studies involving human participants were reviewed and approved by the Ethics Committee of Harbin Medical University. Written informed consent from the participants was not required for them to participate in this study in accordance with the national legislation and institutional requirements.

## Author Contributions

YY and PL conceived and designed the study. AM and ZY collected the data. HC and WW participated in statistical analysis and interpretation of data. ZQ and XQ interpreted the data and wrote the preliminary manuscript. JZ and XY revised the content of the manuscript. PL and JN compiled the data and wrote the preliminary manuscript. All authors contributed to the article and approved the submitted version.

### Conflict of Interest

The authors declare that the research was conducted in the absence of any commercial or financial relationships that could be construed as a potential conflict of interest.
